# Pretreatment prostate specific antigen doubling time as prognostic factor in prostate cancer patients

**DOI:** 10.18632/oncoscience.337

**Published:** 2017-02-24

**Authors:** Gennady M. Zharinov, Oleg A. Bogomolov, Natalia N. Neklasova, Vladimir N. Anisimov

**Affiliations:** ^1^ Department of Radiotherapy, The Russian Research Center of Radiology and Surgical Technologies, St. Petersburg, Russia; ^2^ Department of Carcinogenesis and Oncogerontology, N.N. Petrov Research Institute of Oncology, St. Petersburg, Russia

**Keywords:** prostate-specific antigen, prostate cancer, PSADT, PSA doubling time, education rate

## Abstract

Despite the prostate-specific antigen (PSA) serum level commonly uses as tumor marker in diagnosis of prostate cancer, it seems that PSA doubling time (PSADT) could be more useful indicator of tumor behavior and of prognosis for patients. The results of hormone and radiation therapy were evaluated for 912 prostate cancer having at least 2 PSA tests before the treatment was started. Clustering procedure (selection of homogenous group) was performed by using PSADT as the classification marker. The rate of PSADT was estimated for different dissemination rate, age, Gleasons's score and education level. PSADT index inversely correlated with the rate of prostate cancer dissemination, Gleason's score and the level of education were directly correlated with the age of patients. Survival time was longer and PSADT index was higher in “slow” tumor growing subgroups in local, local-advanced and metastatic prostate cancer patients than these in “fast” subgroups. The study confirmed the prognostic value of pretreatment PSADT in prostate cancer patients independently of cancer progression. No significant relationship exists between the authors and the companies/organizations whose products or services may be referenced in this article.

## INTRODUCTION

Prostate-specific antigen (PSA) – a glycoprotein which is produced and thereby secreted from the epithelium of prostate gland is responsible for liquefaction and ejaculation [1]. Furthermore serum PSA level served as a tumor marker in diagnosis of prostate cancer as well as monitoring of its development. The dynamics of increase in its concentration can be described mathematically in different ways. Among them PSA doubling time (PSADT) is potentially seems most useful. PSADT being sensitive to exponential tumor growth therefore requires a logarithmic analysis [2, 3] Distinctly from PSA index, PSADT can help us learn more about biological behavior of cancer, i.e. to tumor progression [4]. At present time, the estimation of PSADT is recommended for [5]: diagnostic of biochemical relapses after radical treatment with the goal to predict tumor-specific survival rate [6, 7], to select candidates for active observation group [8, 9], as well as in determining the necessity to start treatment of patients who have select active observation.

PSA level without any other clinical data is not considered as a factor in prognosis. Nevertheless it is well known that the pretreatment (primary) PSA level closely correlates with the prostate cancer progression: serum PSA level increase after radical treatment is the main hallmark of tumor recurrence; the dynamics of PSA concentration in response to treatment reflects the effectiveness of therapy [1]. Nevertheless, the rate of PSA kinetics is not considered as the hallmark of tumor growth.

PSADT should be determined before the initiation of treatment in order to obtain information concerning the aggressiveness of prostate cancer, for prediction of treatment results and for monitoring the course of treatment. There are some contradictory data regarding this issue. Some authors proclaim prognostic significance of PSA initial kinetics for disease-free patient survival after beam therapy [10, 11] or radical prostatectomy [12]. However, others authors not support this opinion [13, 14].

The main aim of this study is to evaluate clinical and prognostic significance of PSADT indices, determined before the starting of treatment in patients with prostate cancer, exposed to combined hormone and radiation therapy.

## RESULTS

The PSADT parameters were calculated for 912 prostate cancer patients before starting of their treatment. The number of patients with localized cancer (T1- 2NOMO) was 360 (39.4%) with locally-disseminated (T3- 4NOMO, T1-4N1MO) – 276 (30.3%), with generalized (T1-4NO-1M1) – 276 (30.3%). The mean age of patients was equal to 66.5 ± 7.5 years.

The median value and the interquartile ranges (IQR) of the observation limited by the time of death / control point (which occurred earlier) were estimated as 34.3 (20.2-56.2) months. Median PSADT in the research group – 10.2 (IQR 2.75-36.2) months. Primary PSA level median value was 21.7 (IQR 11.2-53.6) ng/ml.

The data on PSADT parameters as regarded to the tumor process characteristics are presented in the Table 1. In localized cancer group, the median PSADT value was 24.5 (IQR 8.0–69.7) months, in the locally-disseminated prostate cancer – 12.2 (IQR 4.3-36.6) months and in generalized prostate cancer – 2.4 (IQR 1.1– 7.1) months. The differences between the groups were statistically significant (p < 0.00001).

**Table 1:** The PSA-doubling time in patients with different rate of prostate cancer dissemination

Parameters	Number of patients	%	Median PSADT, months (IQR)	p*
*Dissemination*:				
Local	360	39.4	24.5 (8.0 – 69.7)	
Local-advanced	276	30.3	12.2 (4.3 – 36.6)	
Metastatic	276	30.3	2.4 (1.1 – 7.1)	
*Gleason's index*:				< 0.00001
< 6	265	36.4	20.8 (7.4 – 63.4)	
7	242	33.2	9.0 (3.0 – 27.3)	
8-10	222	30.5	3.9 (1.3 – 15.4)	
Primary PSA level, ng/ml				
< 10.0	193	21.2	36.3 (14.4 – 98.1)	
10.1-30.0	357	39.1	13.2 (4.9 – 39.4)	
30.1-100.0	323	25.4	4.5 (1.9 – 17.5)	
> 100.1	130	14.3	1.5 (0.8 – 4.6)	

* ANOVA Kruskal-Wallis test and the median test

Gleason's index was estimated in 729 patients. The median PSADT value in patients with Gleason's index < 6 was 20.8 (IQR 1,3–15,4). In groups with Gleason's index 7 and 8-10 PSADT median values were equal to 9.0 (IQR 3.0-27.3) and 3.85 (IQR 1.3-15.4), respectively. Groups differed significantly from each other (p < 0.00001).

The comparison between PSADT and the primary PSA level also revealed highly significant differences (p < 0.00001). The more PSA level was determined, the less PSADT median value was observed.

The parameters of PSADT in patients of various age and of different education levels are presented in the Table 2. The median PSADT value was 5.5 (IQR 1.4-17.5) months in patients younger than 59 years, 9.0 (IQR 2.5-25.4) months in patients aged from 60 to 69 years; 18.4 (IQR 3.8-52.6) months in patients aged 70-79 months, and 18.6 (IQR 7.6-63.3) months in these older than 80 years.

**Table 2:** The PSA-doubling time in prostate cancer patients of various age and education level

Parameters	Number of patients	%	Median PSADT, months (IQR)	p*
*Age, years*:				
< 59	159	17.4	5.5 (1.4-17.5)	
60-69	404	44.3	9.0 (2.5-25.4)	< 0.01
70-79	322	35.3	18.4 (3.8-52.6)**	
> 80	27	3.0	18.6 (7.6-63.3)	
*Education level:*				
D.Sc.	61	6.7	36.0 (6.0-114.6)	
Ph.D.	75	8.2	22.6 (8.3-92.5)	
University	471	51.6	10.4 (2.9 – 29.5)***	< 0.001
specialized secondary	133	14.6	10.0 (3.3-33.4)	
secondary+ incomplete secondary	172	18.9	3.9 (1.4-20.0)	

* ANOVA Kruskal-Wallis test and the median test

** 70-79 years vs >80 years, p= 0.35.

*** “University” education vs “specialized secondary education”, p=0.67.

The PSADT values were also significantly dependent on the patient's education level (p < 0,001) (Table 2). It was clearly seen that the higher levels of education directly related to the more high parameter of PSADT.

**Figure 1:**
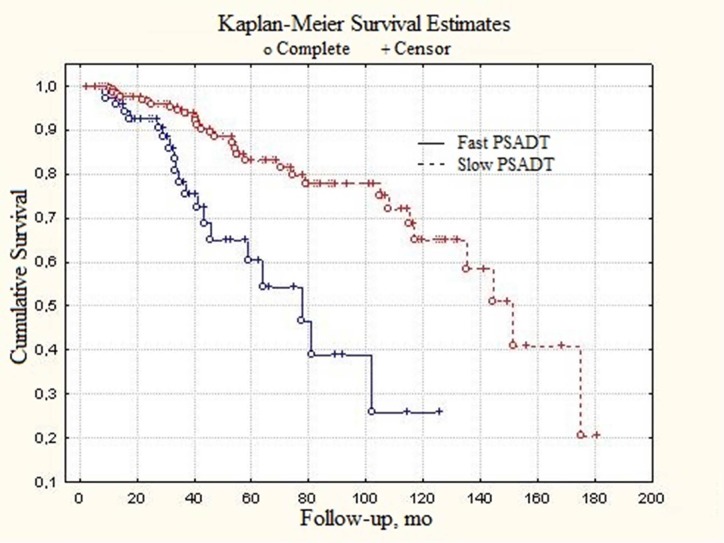
Total survival of local prostate cancer patients depending on PSADT

In order to evaluate the PSADT prognostic value, all patients were divided into subgroups designated as “slow” and “fast” PSADT groups. Parameters characteristic for these groups are presented in the Table 3. The long-rank test shows significant difference between slow and fast subgroups in local prostate cancer patients (Figure 1) (p < 0.01).

**Table 3:** Parameters of PSA-doubling time and survival in prostate cancer patients subdivided according to “slow” and “fast” PSADT value

Parameters	Subgroups by PSADT		P*
	Slow	Fast	
*Local prostate cancer* (n = 360)			
Number of patients	219	141	
Median PSADT (IQR), months	53.1 (29.1 - 119.7)	5.2 (2.8 - 9.3)	< 0.01
Median survival, months	151.2	77.8	Log-rank, p < 0.01
*Local-advanced prostate cancer (n = 276)*			
Number of patients	177	99	
Median PSADT (IQR), months	26.6 (13.2 – 58.5)	3.0 (1.4-4.9)	< 0.01
Median survival, months	Not estimated	69.3	Log-rank, p < 0.01
*Metastatic prostate cancer* (n = 276)			
Number of patients	111	165	
Median PSADT (IQR), months	9.8 (5.2-18.7)	1.3 (0.8 – 2.0)	< 0.01
Median survival, months	49.1	23.4	Log-rank, p < 0.01

* ANOVA Kruskal-Wallis test and the median test

**Figure 2:**
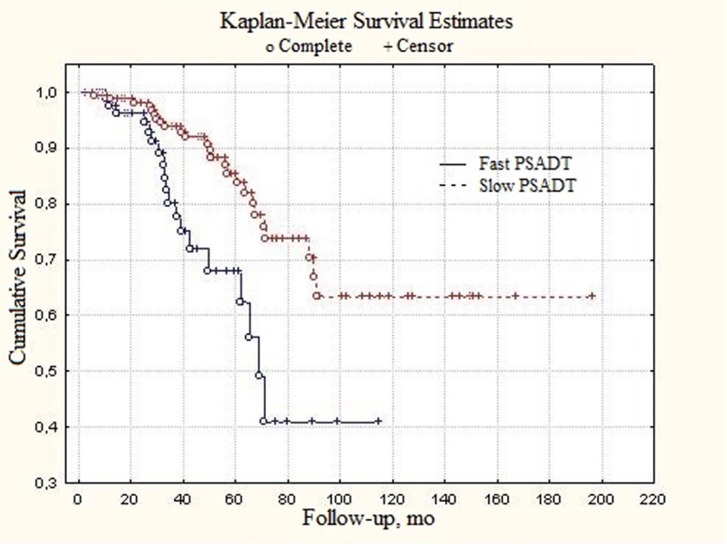
Total survival of local-advanced prostate cancer patients depending on PSADT

**Figure 3:**
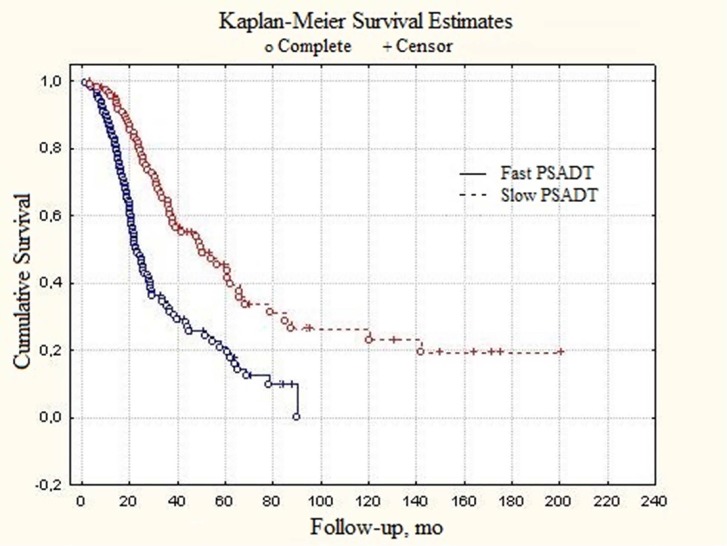
Total survival of generalized (metastatic) prostate cancer patients depending on PSADT

Similar tendencies were found in patients with generalized (metastatic) prostate cancer. Median PSADT values were 23.4 and 49.1 months in fast and slow subgroups, respectively (Figure 2). In patients with locally-disseminated prostate cancer survival curve for slow PSADT did not reach median value level (Figure 3). The difference from the fast PSADT was statistically significant (p < 0.01, log-rank test).

Multivariate analysis revealed that Gleason score (p=0.005), clinical stage (p=0.03) and a pretreatment PSADT (p < 0.001) were independently associated with overall survival. Pretreatment PSA value, hormone therapy timing and duration, and radiation therapy dose were not statistically significant on multivariate or univariate analysis.

## DISCUSSION

The prognostic significance of the primary PSA kinetics in prostate cancer patients, subjected to radical prostatectomy or distant radiotherapy has been discussed in several works [11, 12]. The clinical significance of PSADT level was not evaluated and not compared with the parameters of prostate cancer in both these studies. Only local prostate cancer patients were under observations in both these works. There was no positive association between the initial PSA dynamics and postoperative pathomorphological alterations, as well as patients' survival rate after radical prostatectomy [7]. However, the number of patients being under observation was rather scarce in this study (n=86) as well as postoperative observation time was 2 years, whereas our work based on a much larger cohort of patients (n=912). For the first time the correlation between PSADT with tumor process characteristics, age and patient's education level was demonstrated. We observed that PSADT decreased with the decrease in prostate cancer differentiation. This parameter also decreased with the increase in its dissemination as well as with the rise in primary PSA level. It was established that the PSADT index inversely correlated with the rate of prostate cancer dissemination, Gleason's score and the level of education were and directly correlated with the age of patients. Survival time was longer and PSADT index was higher in “slow” tumor growing subgroups in local, local-advanced and metastatic prostate cancer patients than these in “fast” subgroups. Some authors believe that with increased education level there is a tendency in aggressiveness decrease in prostate cancer [15, 16] which correlates with our data.

Our data suggested that the primary PSADT rate could serve as prognostic factor regardless neoplastic dissemination process. The increase in the rate of PSA correlates with reduction of the prostate cancer patients survival. We believe that primary dynamics of PSA level reflected the rate of prostate cancer growth thus allowing to evaluate it as adequate marker for estimation of prostate cancer growth rate [17, 18].

It was shown that the higher education level or length is associated with greater use of PSA screening and more likely to have a prostate biopsy than men with low (short) education [19–22].

PSA testing without clinical manifestation was also associated with higher education in prostate cancer patients [19]. In our study the PSADT index was higher in patients with local prostate cancer than in patients with metastatic cancer. Also, PSADT was much higher in most qualified (educated) patients than those with only secondary education or less. These data are in agreement with observations demonstrated association between high level of education more healthy lifestyle and more survival rate [22–25].

## MATERIALS AND METHODS

The results of hormone and radiation therapy of 912 patients with prostate cancer were evaluated. All patients were treated and monitored at the Russian Scientific Center of Radiology and Surgical Technologies during the period since 1994 to 2012.

Criteria for including the patients in the research were as follow:

1. Morphologically verified diagnosis of prostate adenocarcinoma;
2. Presence of at least 2 PSA measurements, performed with intervals 1-4 months before starting the treatment.
3. Patient compliance to medications which distort actual PSA value (5α- reductase inhibitors and so on);
4. Positive trend of PSADT value (PSA serum concentration rise during re-examination).

In the selected cohort PSADT was determined before the start of treatment. PSA calculation was made accordance to Memorial Sloan-Kettering Cancer Center recommendations using the PSADT calculator [26].

PSADT clinical significance was determined by comparing this criteria to characteristics of the tumor (spread, sum of notes according to Gleason’s scale i.e. Gleason’s score; PSA primary level – maximal PSA value, based on which biopsy was taken and diagnosis was made), as well as individual patient characteristics (age, education level). The level of education was ranked according to established in Russia system of education as follow: low - secondary school (education length < 11 years;), medium - college for professional education (secondary specialized, 9-12 years), high – university (12-16 years). We included into the analysis scholars with academic degrees - candidates of sciences (practically equal to PhD) and doctors of sciences (DSc). It is commonly assumed that academic degrees reflected more high level of education than university level, graduated from university or, and secondary and incomplete secondary. Beam therapy was performed on the linear accelerators of electrons by bremsstrahlung with limit energy 6 to 18 MeV. Patients with localized prostate cancer received local radiation therapy on target organs (prostate and seminal vesicles) of single local dose 3 Gy, with total local dose 54-57 Gy (equivalent dose: 66-70 Gy). Locally disseminated prostate cancer forms were treated in two steps with daily radiation dose. In the first step of treatment, radiation therapy area except the target including regional lymphatic nodes. Based on documented regional lymph nodes involvement, radiation therapy was performed with single 2 Gy doses up to total 40-44 Gy dose. Step 2 being based on local radiation. Generalized forms of prostate cancer with metastatic pelvic bones involvement were treated with beam therapy starting with segmental radiation step, with single 2 Gy doses up to 20 Gy total, afterwards regional and local radiation was performed according to regimens mentioned above. Patients with generalized metastatic skeletal involvement accompanied with pain syndrome received systemic radiation therapy with ^89^Sr-chloride.

The majority of patients (79%) has been underwent hormone therapy with various gonadotropin releasing hormone analogues and antiandrogen medications. Another 21% of patients were underwent bilateral orchiectomy as hormone therapy method.

Patient were under dynamic observations once every 3 months during the first year and subsequently every 6 months. If it was not possible to observe them on a regular basis or in case of patient's absence at certain times regular phone calls were made, follow up letters were sent to their addresses and consultations with the patient's relatives was requested. Accordingly 1st of July 2013 (control point) the comprehensive information concerning every patient in research group was obtained.

To determine the prognostic significance, correlation between PSADT and total survival was evaluated. Clustering procedure (selection of homogenous group) was performed by using PSADT as the classification marker. To measure similarity the Jaccard index was used and as a part of clustering procedure the mean-K method was used. According to tumor dissemination, patients were divided into subgroups based on slow and fast PSADT indices. Kaplan-Mayer’s survival curves were plotted for each subgroup using the PC program Statistica v.7 (StatSoft Inc., 2002). Difference between the curves was evaluated using log-rank test. To compare two independent selections Mann-Whitney U-test was determined. ANOVA Kruskal-Wallis test and the median test were used for comparing more than two groups. Pretreatment PSA value, Gleason score, tumor stage, timing and duration of hormone therapy, radiation therapy dose, and PSADT were analyzed for any associations with overall survival by using Cox proportional hazards multivariable analysis. With the p-value < 0.05 the difference was considered statistically significant.
